# Influx mechanisms in the embryonic and adult rat choroid plexus: a transcriptome study

**DOI:** 10.3389/fnins.2015.00123

**Published:** 2015-04-28

**Authors:** Norman R. Saunders, Katarzyna M. Dziegielewska, Kjeld Møllgård, Mark D. Habgood, Matthew J. Wakefield, Helen Lindsay, Nathalie Stratzielle, Jean-Francois Ghersi-Egea, Shane A. Liddelow

**Affiliations:** ^1^Department of Pharmacology and Therapeutics, University of MelbourneParkville, VIC, Australia; ^2^Department of Cellular and Molecular Medicine, University of CopenhagenCopenhagen, Denmark; ^3^Walter and Eliza Hall Institute of Medical ResearchParkville, VIC, Australia; ^4^Institute of Molecular Life Sciences, University of ZurichZurich, Switzerland; ^5^Lyon Neuroscience Research Center, INSERM U1028, Centre National de la Recherche Scientifique UMR5292, Université Lyon 1Lyon, France; ^6^Department of Neurobiology, Stanford UniversityStanford, CA, USA

**Keywords:** choroid plexus, brain development, amino acid transfer, protein transfer, brain barriers, cerebrospinal fluid

## Abstract

The transcriptome of embryonic and adult rat lateral ventricular choroid plexus, using a combination of RNA-Sequencing and microarray data, was analyzed by functional groups of influx transporters, particularly solute carrier (SLC) transporters. RNA-Seq was performed at embryonic day (E) 15 and adult with additional data obtained at intermediate ages from microarray analysis. The largest represented functional group in the embryo was amino acid transporters (twelve) with expression levels 2–98 times greater than in the adult. In contrast, in the adult only six amino acid transporters were up-regulated compared to the embryo and at more modest enrichment levels (<5-fold enrichment above E15). In E15 plexus five glucose transporters, in particular Glut-1, and only one monocarboxylate transporter were enriched compared to the adult, whereas only two glucose transporters but six monocarboxylate transporters in the adult plexus were expressed at higher levels than in embryos. These results are compared with earlier published physiological studies of amino acid and monocarboxylate transport in developing rodents. This comparison shows correlation of high expression of some transporters in the developing brain with higher amino acid transport activity reported previously. Data for divalent metal transporters are also considered. Immunohistochemistry of several transporters (e.g., *Slc16a10*, a thyroid hormone transporter) gene products was carried out to confirm translational activity and to define cellular distribution of the proteins. Overall the results show that there is substantial expression of numerous influx transporters in the embryonic choroid plexus, many at higher levels than in the adult. This, together with immunohistochemical evidence and data from published physiological transport studies suggests that the choroid plexus in embryonic brain plays a major role in supplying the developing brain with essential nutrients.

## Introduction

Influx transporters, active across interfaces between the blood and the brain, are essential components of the mechanisms that contribute to the composition and stability of the internal environment of the brain, critical for normal brain function. The main interfaces between these two compartments are the blood-brain barrier across cerebral blood vessels and the blood-cerebrospinal (CSF) barrier across the choroid plexuses within the cerebral ventricles (Saunders et al., 2012). The transport mechanisms in these interfaces have been well studied in the adult brain (Davson and Segal, 1996; Brown et al., 2004; Damkier et al., 2007, 2013; Abbott et al., 2010; Abbott and Friedman, 2012) but relatively little is known about them during gestation, although they are clearly important for normal brain development and maturation. In addition, much of the research focus has been on the blood-brain barrier itself rather than on the transport properties of the choroid plexuses. Nevertheless, in spite of this neglect, it seems that in the early developing brain the choroid plexuses are probably a much more important portal of entry into the brain than are the cerebral blood vessels (Johansson et al., 2008). This is because the choroid plexuses develop to being a substantial and functional tissue at a stage when the brain is still poorly vascularised (Saunders et al., 2012, 2013). By far the largest group of influx transporters is the solute carrier gene series (SLCs), which currently comprises 52 families of 395 transporter genes (Hediger, 2013 and http://www.bioparadigms.org/slc/intro.htm). These are particularly important for ion exchange and amino acid and glucose delivery to the brain. Until recently, very little was known about their expression and function in the choroid plexuses of the developing brain. This paper is the third in a series of combined RNA-Sequencing and microarray analysis of transcriptome expression in developing and adult choroid plexus, with a focus on the plexuses in the lateral ventricles (Kratzer et al., 2013; Liddelow et al., 2013). Here we give a comprehensive analysis of the expression of solute carriers and other influx transporters in developing and adult choroid plexus, with an emphasis on nutrient transporters such as for glucose, amino acids and monocarboxylates and compare these data with available published physiological data on their transport activity *in vivo*. We also include results of immunohistochemical staining of selected SLCs, which confirm that their genes are translating to protein both in the embryo and in the adult. The ion transporters thought to be important for CSF secretion and its homeostasis, as well as brain interstitial fluid have been dealt with in a previous paper (Liddelow et al., 2013) together with the ABC/SLC transporters specifically involved in neuroprotective CSF-to-blood efflux mechanisms (Kratzer et al., 2013). Comparative data are also available from a microarray study of mouse choroid plexus (Liddelow et al., 2012) and an RNA-Seq study of non human primate choroid plexus (Ek et al., 2015).

## Materials and methods

The animal tissue obtained for this study came from two earlier published studies and most methods were as previously described (Kratzer et al., 2013; Liddelow et al., 2013).

### Ethics statement

Animal experiments in Melbourne were conducted in accordance with Australian code of practice for the care and use of animals for scientific purposes 7th Edition, published by the National Health and Medical Research Council. All animal research protocols were reviewed and approved by the University of Melbourne Faculty of Medicine, Dentistry and Health Sciences Animal Ethics Committee and registered under ID. Number 1011703. For experiments conducted in Lyon animal care and procedures were in accordance with the guidelines approved by the French ethical committee (decree 87-848), by the European Community (directive 86-609-EEC).

### Animal husbandry

In Melbourne, timed-pregnant (embryonic day 15, E15) and non-pregnant adult (6 week, 200–300g weight range) Sprague-Dawley rats were used. These ages were chosen as they have been previously shown to be appropriate for studies of the developing lateral ventricular choroid plexus in rodents (Johansson et al., 2006; Liddelow et al., 2012). Animals were supplied by the Biological Research Facility at the University of Melbourne (Victoria, Australia). For next generation RNA-Sequencing, lateral ventricular choroid plexuses from E15 (*n* = 30) and female adult (*n* = 30) rats were used. For immunohistochemistry E15 (*n* = 3) and female adult (*n* = 3) lateral ventricular choroid plexuses were dissected out and processed as described below. For microarray experiments Sprague-Dawley rats (adult males, pregnant time-dated females, or females with their litters) were obtained from Janvier (Le Genest Saint Isle, France). All animals were kept under similar conditions in standard cages, with free access to food and tap water under a controlled environment (12 h day/light cycles).

### Collection of lateral ventricular choroid plexus

The procedure for collection of choroid plexus tissues has previously been described (Liddelow et al., 2012; Kratzer et al., 2013). Briefly, animals were killed by an overdose of inhaled isoflurane (Veterinary Companies of Australia) and brains dissected out under ice-cold RNase-free phosphate buffered saline (PBS, pH 7.3). Both left and right lateral ventricular choroid plexuses were carefully dissected out and placed in fresh ice-cold RNase-free PBS. All steps were performed under RNase-free conditions. The collected tissues were snap-frozen in liquid nitrogen and kept at −80°C until used.

For Illumina RNA-Sequencing, lateral ventricular choroid plexuses from E15 (*n* = 30) and adult (*n* = 30) rats were used (three pooled samples of ten plexuses at each age). Total RNA was extracted using the RNeasy Mini Kit, Qiashredder columns and gDNA removal columns (Qiagen, Valencia, CA, USA) according to standard supplier protocols. All RNA samples were quantified using a NanoDrop ND-1000 UV-VIS spectrophotometer (Thermo Scientific, Wilmington, DE, USA) and quality checked with the Agilent 2100 Bioanalyzer (Agilent Technologies, Palo Alto, CA, USA). Plexuses were pooled (*n* = 10 animals) and centrifuged at 1000 rpm for 30 s, excess PBS removed, snap frozen in liquid nitrogen and stored at −80°C. For Affymetrix microarrays, choroid plexuses were pooled from 3 to 5 animals. Total RNA was isolated from two pools of choroid plexuses sampled from E19, P2, or adult rats using the RNeasy® Micro Kit (Qiagen, Valencia, CA, USA), and DNase-treated according to the manufacturer's protocol.

The choroid plexus consists of epithelium as well as blood vessels, neural innervation and mesenchymal stroma. However, the epithelium is the predominant cell type, suggested to represent up to 90% of the plexus tissue (Keep and Jones, 1990; Liddelow et al., 2012). In this study lateral ventricular choroid plexus was taken *in toto*.

### Illumina next generation RNA sequencing

RNA Sequencing was performed at the Australian Genome Research Facility (Melbourne, VIC, Australia). A cDNA library was prepared from 10 μg of total RNA using the mRNA-Seq Sample Preparation Kit (Illumina, San Diego, CA, USA) according to standard manufacturer protocol. Quality of the library was verified using a DNA 1000 chip using the Agilent 2100 Bioanalyzer (Agilent). The library was subjected to 100 bp single end read cycles of sequencing on an Illumina HiSeq 2000 sequencer as per manufacturer protocol. Cluster generation was performed on a c-Bot (Illumina) with a single read cluster generation kit.

### Data analysis

Short reads were trimmed to remove ambiguous bases from the start and segments with low quality scores from the end, as indicated by the ascii character “B” in Illumina 1.5 phred score encoding. Trimmed reads were mapped with Bowtie version 0.12.7 (Langmead et al., 2009) to the Ensembl (Hubbard et al., 2009) rat genome, release 61. Reads that did not map uniquely were discarded. The number of reads mapped to nuclear genes was determined with HTSeq (Anders, 2010) version 0.4.7p4, using the default “union” counting option. Differential expression between the adult and embryonic samples was detected using an exact test in the Bioconductor (Gentleman et al., 2004) edgeR package, version 2.4.0 (Robinson et al., 2010), with common dispersion used to estimate variance between samples. Genes considered significantly differentially expressed were those with a *p*-value of less than 0.05 after Benjamini-Hochberg false discovery rate correction. All fold changes during development were calculated from these normalized data. However, where appropriate we have also included raw counts for each transcript. As noted in the legends to relevant tables (**Tables 4–6**) ratios for individual genes calculated from raw counts may not correspond to the fold changes (as shown in the tables) calculated from normalized data. We have included both the normalized fold changes and raw cell counts to enable the reader to see both relative expression changes throughout development, as well as the absolute abundance of each transcript at an individual age. A combination of gene ontology annotation and manual curation was used to select genes encoding proteins with integral transporter function. Gene ontology descriptions for rat were downloaded from Biomart (Hubbard et al., 2009), and genes with “transport” mentioned in their gene ontology description were selected. Genes of interest were then extracted from this list. For initial analysis genes with >100 sequence reads and age-related fold changes (FC) > 2.0 (log_2_FC > 1.0) were collated and are summarized in **Tables 4–6**, and Supplementary Tables S1–S3; this level of gene expression was taken as likely to be functionally significant. For identification of the presence of a particular gene a lower cut-off of 10 sequence reads was used; these are also included in **Tables 4–6**. Illumina RNA sequencing data have been deposited with the Gene Expression Omnibus (http://www.ncbi.nlm.nih.gov/geo/) under accession code GSE44072.

### Microarray

As previously described (Kratzer et al., 2013) microarray analysis was performed using a high-density oligonucleotide array (GeneChip Rat Genome 230 2.0 array, Affymetrix, Santa Clara, CA, USA). Total RNA (100 ng) was amplified and biotin-labeled using GeneChip® 3′ IVT Express target labeling and control reagents according to Affymetrix protocol (http://www.affymetrix.com). Before amplification, all samples were spiked with synthetic mRNAs at different concentrations, which were used as positive controls to ascertain the quality of the process. Biotinylated antisense cRNA for microarray hybridization was prepared. After final purification using magnetic beads, cRNAs were quantified using a NanoDrop and quality checked with Agilent 2100 Bioanalyzer. Hybridization was performed according to the Affymetrix protocol. Briefly, 10 μg of labeled cRNA was fragmented and denaturated in hybridization buffer, then hybridized on the chip for 16 h at 45°C with constant mixing by rotation at 60 rpm in a Genechip hybridization oven 640 (Affymetrix). After hybridization, arrays were washed and stained with streptavidin-phycoerythrin (GeneChip® Hybridization Wash and Stain Kit) in a fluidic station 450 (Affymetrix) according to the manufacturer's instruction. The arrays were read with a confocal laser (Genechip scanner 3000, Affymetrix). CEL files summarizing the probe cell intensity data were generated using the Affymetrix GeneChip Command Console (AGCC) software 3.0. Data were normalized with Affymetrix Expression Console software using MAS5 statistical algorithm. Data have been deposited into the Gene Expression Omnibus repository (http://www.ncbi.nlm.nih.gov/geo) under accession number GSE44056.

### Immunohistochemistry

Sagittal sections through E15 and adult rat brain including lateral choroid plexus were selected from the collection of rat tissue at the Faculty of Health and Medical Sciences, University of Copenhagen and used for immunohistochemical detection of SLC16a10, Slc38a5 (SNAT5), Slc4a1, Slc11a1 (NRAMP), Slc39a4 (ZIP4), and Slc1a3 (EAAT1). Sections were deparaffinised in xylene, rehydrated through graded alcohols followed by treatments in 0.5% hydrogen peroxide in methanol for 15 min and rinsing in TRIS buffered saline (TBS) as described previously (Liddelow et al., 2012). Following removal of non-specific binding by incubation for 30 min with blocking buffer (ChemMate antibody diluent S2022, DakoCytomation, Glostrup, Denmark) at room temperature sections were incubated in primary antibodies as listed in Table 1.

**Table 1:** **List of primary antibodies used for immunohistochemistry**.

**Primary antibody**	**Host IgG**	**Dilution**	**Retrieval**	**Producer**	**Code number**
Slc16a10 (MCT10)	Rabbit	1:20	M6	Abcam	Ab121519
Slc38a5 (SNAT5)	Goat	1:150	-	Santa Cruz	Sc-50682
Slc4a1 (AE1)	Rabbit	1:100	-	Proteintech	18566-1-AP
Slc11a1 (NRAMP)	Rabbit	1:70	-	Santa Cruz	Sc-20113
Slc1a3 (EAAT1)	Rabbit	1:600	-	Abcam	Ab416
Slc5a5 (NIS)	Rabbit	1:200	-	Proteintech	24324-1-AP
SLC 39a4 (ZIP4)	Rabbit	1:200	-	Proteintech	20625-1-AP

After overnight incubation sections were washed in TBS and incubated for 30 min in EnVisionTM+ System/HRP (DAKO), K5007, for rabbit antibodies and RPN1025 from GE Healthcare for donkey anti-goat antibodies and then Vector: Vectastain RTU elite ABC reagent PK 7100. This was followed by 6 min incubation with DAB-chromogen solution (DAKO) and counterstaining with Mayer's haematoxylin, dehydrated and mounted with DPX. Control sections contained no primary antibodies and were always blank.

### Photography and image preparation

Digitized images were obtained using an Olympus DP70 camera housing (Olympus, Tokyo, Japan) attached to an Olympus BX50 light microscope (Olympus). A10× eyepiece and 40× objective lens were used. Raw image files were process in Adobe Photoshop CS5® (Adobe® Systems, San Jose, CA, USA). The brightness and curve functions were used to obtain images with background close to white. There was no other manipulation of images.

## Results

### Solute carriers (SLCs) identified by RNA-sequencing and microarray

**Figure 1:**
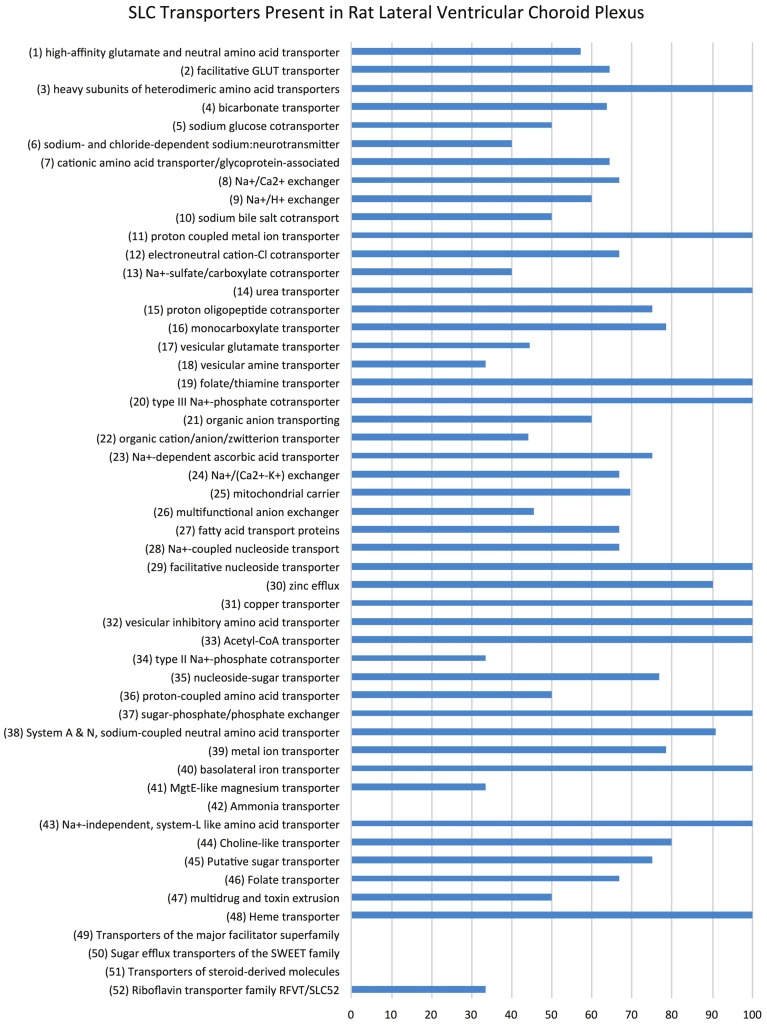
***Slc* transporter genes identified in rat lateral ventricular choroid plexus**. Expression of SLC family class transcripts in the rat lateral ventricular choroid plexus regardless of age, as determined by RNA sequencing. The 52 known functional groups of SLC transporters (Hediger, 2013) are shown on the left. Data are expressed as percentage total of each family of transporters (transcript number > 10)—for example, 100% of proton-coupled metal ion transporters (SLC11 family) are present in the rat lateral ventricular choroid plexus, whereas no steroid-derived molecule transporters (SLC51 family) were detected.

**Figure 2:**
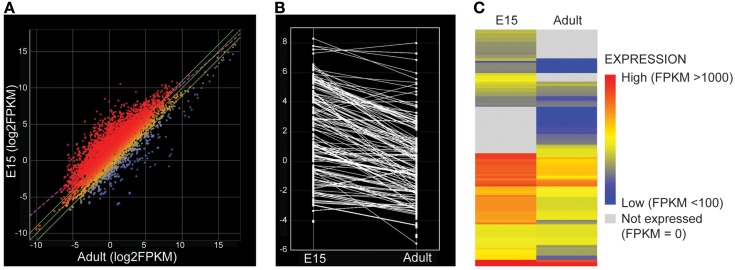
**(A)** Scatter plot comparing log2(FPKM), i.e., fragment count per kilobase of exonic length per million reads mapped, for all transcripts detected by RNA sequencing of the rat lateral ventricular choroid plexus, averaged across replicates from embryonic day 15 (E15) and adult. Red points show transcripts with higher expression at E15 (greater than 2-fold) while blue points are those with higher expression in the adult. FPKM was estimated using a standard ArrayStar pipeline (http://www.dnastar.com/t-help-arraystar.aspx). There is a greater number of transcripts enriched in the embryo. **(B**) Parallel coordinate plot of change in average expression (log2(FPKM)) of SLC transporters in E15 and Adult lateral ventricular choroid plexus. Lines connect the same transcript present at both ages. **(C)** Heat map of average expression (log2(FPKM)) for SLC transporters. Red depicts high expression values, blue low expression. Gray depicts transcripts that were not detected at one age. The expression level of SLC transporters was much higher at E15—as shown by more red expression data.

**Figure 3:**
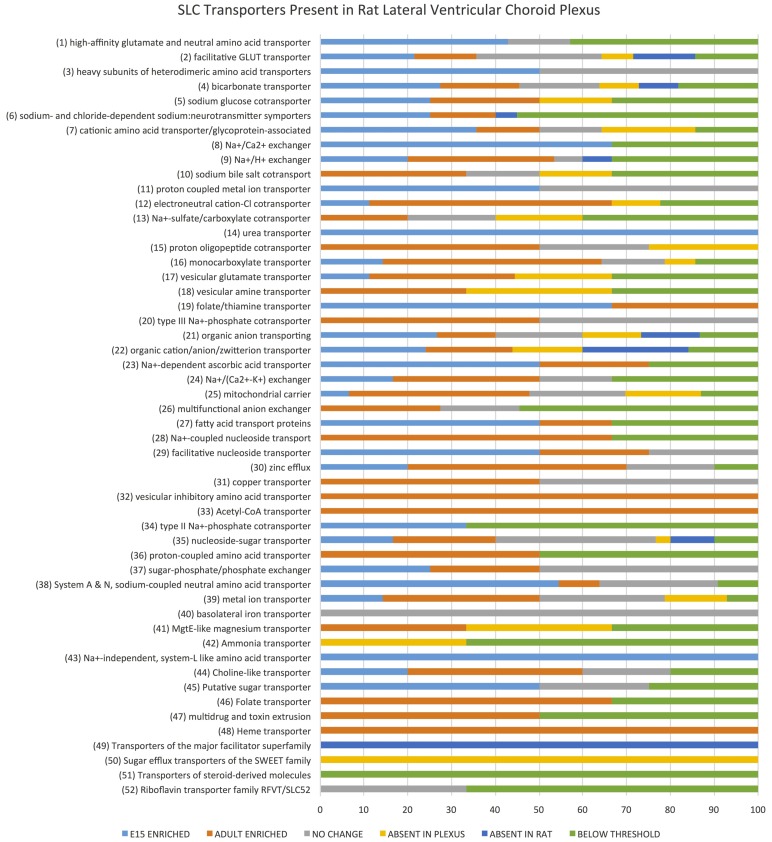
***Slc* transporter genes identified in rat lateral ventricular choroid plexus**. Expression of SLC family class transcripts in the embryonic and adult lateral ventricular choroid plexus as determined by RNA sequencing. Data are expressed as percentage total of each family of transporters. The 52 known functional groups of SLC transporters (Hediger, 2013) are shown on the left. The horizontal bars show the percentage of total number of members of each group identified “Enriched” refers to transcripts with greater than 2-fold expression in one age over the other, while “no change” refers to transcripts with similar expression at both ages. A transcript was considered absent in the choroid plexus if no single copy of transcript was detected (while those with “below threshold” had raw count values below 10). A transcript was considered absent in the rat if no annotation was found in the online ensemble database (http://www.ensembl.org/Rattus_norvegicus).

Examination of the whole transcriptome data set from RNA-Sequencing showed 48 families of the 52 known SLC families (Hediger, 2013) and 64% of transcripts in these families were identifiable at functionally significant levels of expression (>100 transcript reads, Figure 1, Supplementary Table S1). The distribution of genes at the two ages that showed increased, decreased or unchanged expression is illustrated in Figure 2. For each SLC family the distribution of gene expression in embryonic and adult choroid plexus is shown in Figure 3.

The *Slc* genes that were expressed at a higher level (>2 fold) in the embryonic choroid plexus than in the adult are listed in Table 2; microarray data from E19 and P2 are also shown. The *Slc* genes that were expressed at a higher level (>2 fold) in the adult plexus are shown in Table 3; data comparing adult with E19 and P2, from microarray, are also shown. The main focus of this report is the *Slc* transporter genes involved in nutrient transport into CSF, particularly amino acids, monocarboxylates and glucose.

**Table 2:** **Expression and function of solute carrier (Slc) transcripts that were enriched in lateral ventricular choroid plexus of rat E15 and E19 embryos and P2 neonates compared to adult**.

**Gene ID**	**Protein**	**Transport function**	**Ref.**	**E15/A**	**E19/A (E19/P2)**	**P2/A**
*Slc16a10*	MCT10	Aromatic AAs, T3, T4	1	E	P (2.1)	P
*Slc38a5*	SN2, JM24,	Na^+^-dependent AAs	2	E	P (0.6)	P
*Slc43a3*	EEG1	Na^+^-independent, L-like AAs	2	E	−	−
*Slc39a10*	ZIP10	Zn^2+^		E	−	−
*Slc35f1*	FLJ13018	Nucleoside-sugar		60	−	−
*Slc4a1*	AE1, CD233	Anion (Cl^−^-HCO_3_ exchange)	6, 7	59	6.4	3.4
*Heph*	Hephaestin	Fe^2+^, Cu^2+^		E	6.1	6.3
*Slc7a11*	xCT	Cysteine, glutamate	2, 5	44	24	5.4
*Slc30a2*	ZnT2	Zn^2+^	9	E	P (1.7)	P
*Slc14a2_v1*	UTR, UT2	Urea transport		39	−	−
*Slc39a8*	ZIP8	Zn^2+^, Cd^2+^	9	37	P (1.3)	P
*Slc6a4*	SERT	Na-dependent 5HT transport		35	−	−
Slc30a10^*^	ZnT10	Zn^2+^		E	−	−
*Slc43a1*	LAT3	Neutral AAs (phenylalanine)	2	E	P (0.8)	P
*Slc35f2*	FLJ13018	Nucleotides into Golgi		19	−	−
*Slc7a1*	CAT1	Cationic AAs (arginine, lysine)	2, 5	16	11	3.7
*Slc8a3*	NCX3	Na^+^/Ca2^+^ exchanger		15	−	−
*Slc38a1*	NAT2	Glutamine (Na^+^-dependent)	3	15	P (1.0)	P
*Slc6a6*	TAUT	Taurine, β-alanine	2, 5	14	6.9	6.1
*Sfxn1*	Sideroflexin 1	Fe^2+^		14	8.1	7.2
*Slc6a15*	BOAT2	Neutral AAs	2, 5	14	9.8	4.3
Slc22a6^*^	OAT1, NKT	Organic anions (Na^+^-dependent), kynurenic acid, Hg^2+^		14	−	−
*Slc23a1*	SVCT1	Na dependent ascorbic acid		13	−	−
*Slc39a11*	ZIP11	Zn^2+^ transporter		12	−	−
*Slc2a3*	GLUT3	Glucose transporter		11	0.8	0.7
*Slc5a7*	CHT	Choline, Na^+^/glucose co-transport		11	−	−
*Slc17a6*	VGLUT2	Glutamate	3	9.1	−	−
*Slc7a3*	CAT3	Cationic amino acids	2, 5	9.1	P (1.0)	P
*Slc11a1*	NRAMP	Fe^2+^, Mn^2+^, arginine	10, 11	E	1.6	1.4
*Slc8a1*	NCX1	Na^+^/Ca^2+^ exchanger		8.1	−	−
*Slc27a6*	FATP6	Long chain fatty acids		7.8	−	−
*Slc45a3*	PROSTEIN	Putative sugar transporter		6.6	−	−
*Slc34a2*	NaPi-IIb	PO^3−^_4_ (Na^+^-dependent)		6.1	P (1.4)	P
*Slc19a3*	THTR2	Thiamine		6.0	−	−
*Slco2a1*	PGT, OATP2A1	Prostaglandin D2, E1, E2 and F2A, organic anions		5.7	P (1.7)	P
*Slc38a11*	−	Na^+^-dependent AA/proton antiporter (putative)		5.3	−	−
*Slc5a8*	SMCT1	Monocarboxylate transporter		5.2	−	−
*Slc16a14*	MCT14	Monocarboxylate transporter		5.2	−	−
*Slc38a4*	ATA3	Acidic and neutral AAs	2. 3, 5	5.2	−	−
*Slc35d2*	HFRC	Nucleotide sugars		5.1	−	−
*Slc1a4*	SATT	Glutamate, neutral AAs alanine, serine, cysteine, threonine	3, 4	5.1	−	−
*Slc1a3*	GLU-T	Glutamate, neutral AAs	3, 4	4.5	3.3	2.8
*Slc6a13*	GAT3	GABA transporter	8	4.4	P (0.4)	P
*Slc9a5*	NHE5	Na^+^/ H^+^ exchanger	6, 7	4.4	P (0.9)	P
*Slc37a2*	SPX2	Glucose-6-phosphate antiporter		4.2	−	−
*Slc4a4*	NBC1	Electrogenic NaHCO_3_	6, 7	3.9	−	−
*LOC100360087*	Ferritin Light Chain, Flt1-like	Fe^3+^ storage		3.6	2.4	1.7
*Slc22a2*	OCT2	Organic cations, anions, zwitterions		3.4	−	−
*Slc29a2*	ENT2	Nucleosides		3.2	1.2	1.1
*Trfr*	TFR	Transferrin receptor		3.1	0.5	0.6
*Slc2a1*	GLUT1	Glucose		3.0	1.9	0.9
*Slc27a3*	FATP3	Long-chain fatty acids		2.9	2.3	2.9
*Slc24a6*	−	Na^+^/Li^2+^/Ca^2+^ exchanger		2.8	1.2	1.2
*Slc7a2*	CAT2	Low affinity cationic AAs		2.7	P	−
*Slc4a3*	AE3	Anion exchange	6, 7	2.7	0.6	0.8
*Slc25a27*	UCP4	Mitochondrial uncoupling protein		2.7	−	−
*Slc38a7*	SNAT7	Putative Na^+^-coupled neutral AAs		2.7	−	−
*Slc22a15*	FLIPT1	Organic cations, anions, zwitterions		2.6	2.1	1.8
*Slc35e1*	FLJ14251	Nucleotide sugars		2.5	1.2	0.9
*Slc22a9*	OAT7	Organic anions		2.5	−	−
*Slc14a1*	UT-B1-B2	Urea		2.4	−	−
*Slc22a23*	−	Organic cations, anions, zwitterions		2.4	1.7	1.0
*Slc43a2*	LAT4	Large neutral AAs		2.4	1.3	1.4
*Slco2b1*	OATP2B1	Organic anions		2.4	2.3	2.3
*Slc29a3*	ENT3	Nucleosides		2.1	0.8	0.7
*Slc1a5*	ASCT2	Neutral AAs	3,4	2.1	0.8	0.7

*E15 from RNA-Seq (Only transcripts with fold change (FC) > 2.0, p value < 0.05, and raw Illumina counts >100 are shown; actual counts are in **Tables 4–6**). E19 and P2 data from microarray. -: not on the microarray or not detected. P indicates that a signal was detected in E19 or P2, but was below background level in adult samples. The fold change between E19 and P2 is shown in parenthesis. E indicates that a signal was detected in E15 but was below threshold in adult samples*.

* *transporter suggested to be involved in efflux of molecules out of the CSF. A comprehensive list with gene IDs and gene descriptions and statistical data is in Supplementary Table S2 of Supplementary Material. Some genes that are not Slc but play a role in blood-CSF transport of metals, have also been added to this table. Ref indicates publications with information on amino acid transport: ^1^Porterfield and Hendrick (1992); ^2^Lefauconnier and Trouvé (1983); ^3^Al-Sarraf et al. (1997); ^4^Braun et al. (1980); ^5^Cornford et al. (1982); ^6^Brown et al. (2004); Adult ^7^Damkier et al. (2007) Adult, ^8^Al-Sarraf (2002); ^9^Chowanadisai et al. (2005); ^10^Moos and Morgan (1998); ^11^Morgan and Moos (2002)*.

**Table 3:** **Expression and function of solute carrier (Slc) transcripts that were enriched in lateral ventricular choroid plexus of adult rats compared to E15 RNA-Seq) and E19 and P2 (microarray)**.

**Gene ID**	**Protein**	**Transport function**	Distribution^**^	**A/E15**	**A/E19**	**A/P2**
*Tf*	Transferrin	Fe^2+^	ubiquitous	183	5.1	2.3
*Lcn2*	Lipocalin 2	Fe^2+^	ubiquitous	55	6.2	1.0
*Slc16a8*	MCT3	Monocarboxylate	brain	55	−	−
*Slc6a20*	XTRP3	Na^+^- and Cl^−^-dependent AAs	apex epithelial cells (gut)	24	1.0	1.1
*Slc29a4*	ENT4	monoamines	brain	19	−	−
*Slco1c1*	OATP1C1	organic anions, T4, T3	BBB	18	5.3	3.2
*Slc9a2*	NHE2	Sodium/hydrogen exchanger	BBB	18	a	1.3
*Slc21a4*	OAT-K1	organic anions	not brain or CP	14	−	−
*Slc44a3*	CTL3	Choline	not brain or CP	14	−	−
*Slc25a31*	PHC	ADP/ATP	brain	14	−	−
*Slc24a3*	NCKX3	Na/K/Ca exchanger	CP^*^	12	a	2.3
*Slc39a12*	ZIP12	zinc	brain	12	−	−
*Slc19a2*	ThTr1	thiamine	ubiquitous	11	3.6	2.3
*Slc28a3*	CNT3	purine, pyrimidine nucleosides	not brain or CP	10	−	−
*Slc17a8*	VGLUT3	Vesicular glutamate	brain	8.9	−	−
*Slc12a3*	NCC	K+/Cl2 co-transporter	CP^*^	7.5	−	−
*Slc12a2*	NKCC1	Na^+^/K^+^/Cl^−^2	CP^*^	7.5	1.6	1.4
*Slc5a11*	SMIT2	Myoinositol	brain	6.6	−	−
*Cp*	Ceruloplasmin	Cu^2+^ binding	ubiquitous	6.2	0.6	0.3
*Slc22a25*	UST6	unknown	liver	6.0	1.9	1.0
*Slc24a5*	NCKX5	Na^+^/Ca^2+^/K^+^ coupled	brain	5.8	1.1	1.4
*Slc17a9*	VNUT	vesicular nucleotide	brain	5.8	−	−
*Slc4a5*	NBCe2	Electrogenic NaHCO3 cotransporter	CP^*^	5.4	−	−
*Slc35d1*	UGTREL7	UDP-glucuronic acid/UDP-N-acetylgalactosamine	ubiquitous	5.2	−	−
*Slc22a5*	OCTN2	zwitterions, organic cations	not brain or CP	5.1	1.8	1.4
*Slc2a12*	GLUT12	glucose	not brain or CP	5.1	−	−
*Slc31a1*	CTR1	copper	ubiquitous	4.7	1.3	1.1
*Slc35a1*	CST	CMP-sialic acid transporter	ubiquitous	4.5	2.0	1.5
*Slc6a8*	CT1	Na- and Cl-dependent creatine	brain	4.5	2.0	1.8
*Slc3a1*	rBAT	Neutral and basic amino acids	not brain or CP	4.5	0.6	0.3
*Slc30a4*	ZNT4	Zinc	brain	4.5	1.3	1.1
*Slc23a2*	SVCT2	ascorbic acid	epithelial tissues	4.2	−	−
*Slc7a4*	CAT4	cationic amino acids	brain	4.0	1.4	1.5
*Slc39a13*	ZIP13	Zinc	ubiquitous	3.9	2.3	1.7
*Slc17a5*	SIALIN	acidic sugar	ubiquitous	3.8	1.5	1.6
*Slc25a16*	GDC	Graves disease carrier protein	brain	3.8	1.9	1.5
*Slc10a6*	SOAT	organic anions, primarily sulphated steroids	not brain or CP	3.8	NOA	NOA
*Slc30a3*	ZNT3	Zinc	brain synaptic vesicles	3.8	2.6	1.9
*Slc16a12*	MCT12	proton-linked monoarboxylate	not brain or CP	3.8	−	−
*Slc16a6*	MCT7	monocarboxylate	brain	3.7	1.9	1.7
*Slc25a29*	ORNT3	Mitochondrial carnitine/acylcarnitine	brain	3.5	1.7	1.1
*Slc18a2*	VMAT2	5-HT, DA, AE, adrenalin, histamine	CNS aminergic	3.4	−	−
*Slc19a1*	RFC	Folate	ubiquirous	3.4	1.3	1.4
*Slc32a1*	VIAAT	Vesicular inhibitory Aas, GABA/Glycine	CNS	3.4	−	−
*Slc25a40*	−	unknown	ubiquitous, low levels	3.4	−	−
*Slc30a5*	ZNT5	Zinc	not brain or CP	3.4	2.0	1.9
*Slc9a7*	NHE7	sodium/hydrogen exchanger	CP^*^	3.4	−	−
*Slc25a46*	−	unknown	ubiquitous	3.3	1.2	1.0
*Slc46a3*	−	unknown	unknown	3.3	1.5	1.5
*Slc4a2*	AE2	Anion exchange	CP^*^	3.3	2.7	2.9
*Slc5a6*	SMVT	Sodium-dependent multivitamin, biotin	brain	3.2	1.7	1.5
*Slc25a17*	PMP34	peroxisomal membrane protein	brain	3.1	1.6	1.5
*Slc15a3*	PHT2	di- and tri-peptides	“faintly” in brain	3.1	−	−
*Slc12a4*	KCC1	K^+^/Cl^−^	CP^*^	3.1	2.2	2.0
*Slc10a4*	P4	Sodium/bile acid cotransporter	brain (cholinergic neurons)	3.0	−	−
*Slc25a12*	AGC1	L-glutamate, aspartate	brain	3.0	1.9	1.8
*Slc25a3*	PHC	Phosphate, mitochondrial	brain	2.9	1.3	1.2
*Slc26a3*	DRA/CLD	Chloride anion exchanger	not brain or CP	2.9	−	−
*Slc25a4*	ANT1	ADP/ATP	brain	2.9	1.1	1.1
*Slc15a2*	PEPT2	Oligopeptides, antibiotics	choroid plexus apical surface	2.8	0.7	0.8
*Slc25a5*	ANT2	ADP/ATP	brain	2.8	1.2	1.2
*Slc38a3*	SNAT3	Sodium-coupled neutral amino acids	not brain or CP	2.7	1.2	1.2
*Slc25a14*	UCP5	Brain mitochondrial carrier protein	highest in brain and testis	2.7	1.6	1.4
*Slc30a6*	ZNT6	Zinc	brain	2.7	1.3	1.1
*Slc22a18*	−	Probably organic anions	not brain or CP	2.6	1.4	1.6
*Slc37a1*	SPX1	Glycerol-3-phosphate	ubiquitous	2.6	2.1	1.4
*Slc25a35*	−	unknown	unknown	2.6	−	−
*Slc33a1*	ACATN1	Acetyl-coenzyme A	brain	2.6	1.4	1.3
*Slc36a1*	PAT1	Neutral amino acids, glycine, proline, GABA	brain	2.4	−	−
*Slc16a1*	MCT1	Monocarboxylates	ubiquitous	2.4	3.1	1.4
*Slc20a1*	PIT1	Sodium-dependent phosphate	ubiquitous	2.4	1.7	2.0
*Slc36a4*	PAT4	Proton-coupled AAs, tryptophan, proline	ubiquitous	2.3	−	−
*Slc48a1*	HRG1	Heme transporter	brain	2.3	1.9	1.3
*Slc25a32*	MFT	Mitochondrial folate	unknown	2.3	1.1	1.0
*Slc41a1*	MgtE	Mg^2+^, other metal cations	not brain or CP	2.3	a	1.7
*Slc16a13*	MCT13	Monocarboxylate	not brain or CP	2.3	1.9	1.7
*Slc26a4*	PENDRIN	Anions	epithelial cells	2.2	−	−
*Slc16a2*	MCT8	Monocarboxylate transporter 8, thyroxine	brain	2.1	−	−
*Slc25a20*	CACT	Mitochondrial carnitine/acylcarnitine	brain	2.1	1.1	0.9
*Slc35b3*	PAPST2	Adenosine 30-phospho 50-phosphosulphate	ubiquitous	2.1	1.7	1.5
*Slc7a10*	ASC1	Asc-type amino acids, D-serine	brain	2.1	0.6	0.8
*Slc31a2*	CTR2	Cu^2+^	ubiquitous	−	1.7	1.0

For RNA-Seq) only transcripts with fold change (FC) > 2.0, p value < 0.05, and raw Illumina counts > 100 are shown. A comprehensive list with gene IDs and gene descriptions and statistical data is in Table S2 of Supplementary Material. For microarrays: - : not on the array or not detected. a: absent in E19 samples. CP^*^: choroid plexus (Liddelow et al., 2013).

** *Previously described tissue distribution from reviews in Hediger (2013). Note that in studies involving brain extraction it is often not stated if choroid plexuses were included or excluded*.

In the present study the numerous members of the SLC families of transport proteins that were present in the lateral ventricular choroid plexus at E15 and in the adult had strikingly different patterns of gene expression at these two ages: of 250 total genes identified by RNA sequencing (Supplementary Table S2), 81 were expressed at higher levels at E15 (Supplementary Table S2 and Table 2). Developing-to-adult fold change differences between E19 and P2 were modest, apart from *Slc7a11*, *Slc7a1*, and *Slc6a15*, suggesting that birth is not a key milestone for developmental changes in the choroidal Slc transcriptome. A number of genes such as *Slc4a1, Slc2a3, Slc11a1* had strikingly higher E15/Adult fold change than E19-P2/Adult fold changes. This suggests very specific choroidal function for brain development at the early stages of gestation. 107 genes were up-regulated in the adult (Supplementary Table S3 and Table 3). For these genes the array analysis shows that the E19/Adult and P2/Adult fold changes were modest and lower than E15/Adult fold changes for most of these genes, indicating that the choroid plexus starts to acquire its “adult” phenotype early in development, i.e., at late prenatal stages in the rat. Finally, 62 genes showed no difference in expression level between the ages (Supplementary Table S3), indicating the early maturity and life-long duration of different choroidal functions, in addition to early development-specific functions assumed from the E15 specific genes identified. Many fewer *Slc* transporter genes have been identified at the blood-brain barrier (Daneman et al., 2010—supplementary material) of about 56 transcripts, which is around a quarter of that identified in this choroid plexus study. This finding supports the previously suggested proposition that in the developing brain the choroid plexus is the main route of entry for amino acid and ion transport and other molecules essential for brain development (Johansson et al., 2008).

#### Glucose and monocarboxylate transporters

Table 4 lists the glucose and monocarboxylate transporters differentially regulated in the embryo and in the adult with a fold change (FC) of two or more. *Slc5a10* (sodium/glucose co-transporter) was expressed only in the embryonic plexus, but at a low copy number. Gene array shows that it was still expressed at E19, but disappeared after birth. The number of glucose transporters expressed at a higher level in the embryo was strikingly more than those higher in the adult (five compared to two, note that Klf15 is a regulator of GLUT4, not a transporter itself, Gray et al., 2002). The converse was the case for monocarboxylate transporters; there was only one in the embryo, but 6 in the adult.

**Table 4:** **Glucose and Monocarboxylate transporters in developing rat choroid plexus**.

**Gene name**	**Gene description**	**FC**	**Raw transcript counts**	
			**EMBRYO (E15)**	**ADULT**
**GLUCOSE TRANSPORTERS**				
**Embryo**		**E/A**		
*Slc5a10*	Slc family 5, sodium/glucose cotransporter 10	E	62	0
*Slc2a3*	Slc family 2, facilitated glucose transporter 3, GLUT3	E	150	8
*Slc2a4*	Slc family 2, facilitated glucose transporter 4, GLUT4	E	90	6
*Slc35d2*	UDP-N-acetylglucosamine/UDP-glucose/GDP-mannose	5.1	224	25
*Slc45a1*	Proton-associated sugar transporter A, glucose	E	33	5
*Slc2a1*	Slc family 2, facilitated glucose transporter 1, GLUT1	3.0	368	111
**Adult**		**A/E**		
*Slc2a12*	Slc family 2, facilitated glucose transporter 12, GLUT12	5.1	3569	10437
*Slc2a5*	Slc family 2, facilitated glucose transporter 5, GLUT5	2.3	20	27
*Klf15*	Krueppel-like factor 15, regulator of GLUT4	2.2	292	373
**MONOCARBOXYLATE TRANSPORTERS**				
**Embryo**		**E/A**		
*Slc16a10*	Monocarboxylate transporter 10, MCT10, TAT1, T3, T4	E	3089	51
**Adult**		**A/E**		
*Slc16a8*	Monocarboxylate transporter 3, MCT3	55	49	1524
*Slc16a6*	Monocarboxylate transporter 7, MCT6, MCT7	3.7	1524	3239
*Slc16a1*	Monocarboxylate transporter 1, MCT1	2.4	577	1235
*Slc16a7*	Monocarboxylate transporter 2, MCT2	2.3	44	58
*Slc16a13*	Monocarboxylate transporter 13, MCT13-human	2.3	1354	1756
*Slc16a2*	Monocarboxylate transporter 8, MCT8-human, thyroid hormone	2.1	7599	9323

*RNA-Sequencing expression levels of glucose and monocarboxylate transporters in the E15 and adult rat lateral ventricular choroid plexus. Fold change data obtained from normalized data and refer to expression change compared to opposite age. Raw count data have not been normalized and thus the calculated fold changes EC may be different from E/A raw count ratios. E is embryo only. A is adult only*.

*Slc2a3* (GLUT3) was only detected in the embryo using RNA-Seq, but in microarray data was expressed at similar levels in the adult (ratio of 0.7–0.8). However, the transcript numbers in RNA-Seq were very low, so this result is of dubious functional significance. Three other glucose transporters (*Slc2a4, Slc2a5, Slc45a1*) had transcript numbers <100 at both ages and were not detected in microarray, so these genes are unlikely to be contributing to glucose transport via the choroid plexuses. More relevant is GLUT1 (*Slc2a1*) which is well expressed in embryonic and E19 fetal choroid plexus relative to P2 or adult. This transporter is crucial for glucose delivery to the brain. Its expression in the developing brain choroid plexus argues for a specific role of the plexus in nutrient transport at these ages. Amongst monocarboxylate transporters the same single one (*Slc10a16*) was increased in the developing choroid plexus in both RNA-Seq and microarray results. The most highly expressed of all the monocarboxylate transporters (in terms of transcript numbers) was *Slc16a2*, at a slightly higher (2.1 fold) level in the adult than in the embryo; this transporter was not detected in microarray, possibly because the probe was not a good match. *Slc16a8* was expressed at a high level in adult plexus, but expression was <100 transcript counts in the embryo in the RNA-Seq analysis and was also not detected in microarray. Three other monocarboxylate transporters were expressed at high level in adult plexus in both microarray and RNA-Seq (Tables 3, 4).

#### Amino acid transporters

There were twice as many genes coding for amino acid transporters in the embryonic choroid plexus expressed at a higher level than in the adult (12 compared to 6, Table 5). The level of expression of these transporters in the embryo was also generally higher; thus in the embryo nine of the transporters were expressed at >5 fold compared to adult, whereas in the adult only one transporter gene was expressed >5 fold compared to the embryo. The microarray results were generally consistent with those from RNA-Seq (Tables 2, 4, 5). For both methods the expression levels of *Slc7a11, Slc7a1*, *Slc6a6*, and *Slc6a15* were notably high in the embryonic plexus. For two genes (*Slc3a1*, *Slc7a10*) the expression levels were higher in the adult with RNA-Seq, but lower with microarray (Tables 3, 5).

**Table 5:** **Expression of amino acid transporters in embryonic (E15) and adult rat choroid plexus**.

**Gene name**	**Gene description**	**FC**	**Raw transcript counts**	
			**EMBRYO (E15)**	**ADULT**
**AMINO ACID TRANSPORTERS**				
**Embryo**		**E/A**		
*Slc38a5*	Sodium-coupled neutral amino acid transporter 5, glycine	E	1021	6
*Slc43a3*	Putative transporter (by similarity)	E	914	8
*Slc7a11*	Solute carrier family 7 (cationic amino acid transporter, y+ system), member 11	44	13298	178
*Slc43a1*	Large neutral amino acids transporter small subunit 3	E	204	5
*Slc7a1*	High affinity cationic amino acid transporter 1	16	7273	264
*Slc38a1*	Sodium-coupled neutral amino acid transporter 1, glutamine	15	1188	47
*Slc6a6*	Sodium- and chloride-dependent taurine transporter	14	813	33
*Slc6a15*	Orphan sodium- and chloride-dependent neurotransmitter transporter NTT73	14	5446	227
*Slc7a3*	Cationic amino acid transporter 3, l-lysine, l-ornithine, l-arginine	9.1	42.0	2.7
*Slc38a4*	Sodium-coupled neutral amino acid transporter 4	5.2	102	11
*Slc1a4*	Neutral amino acid transporter A, l-serine, l-alanine, l-cystine, l-proline,	5.1	436	49
*Slc1a3*	Excitatory amino acid transporter 1, l-glutamate transport, malate-aspartate	4.5	4372	577
*Slc7a2*	Low affinity cationic amino acid transporter 2, l-arginine, l-lysine, l-ornithine	2.7	179	38
*Slc38a7*	Putative sodium-coupled neutral amino acid transporter 7	2.7	337	73
*Slc43a2*	Large neutral amino acids transporter small subunit 4	2.4	651	156
*Slc1a5*	Neutral amino acid transporter B(0) l-serine,	2.1	1119	308
**Adult**		**A/E**		
*Slc6a20*	Sodium- and chloride-dependent transporter XTRP3, proline IMINO transporter	24	44	594
*Slc6a11*	Sodium- and chloride-dependent GABA transporter 3	5.0	10	28
*Slc3a1*	Neutral and basic amino acid transport protein rBAT	4.5	746	1936
*Slc7a4*	Cationic amino acid transporter 4	4.0	706	1618
*Slc38a3*	Sodium-coupled neutral amino acid transporter 3. l-asparagine, l-glutamine, l-histidine	2.7	4507	6922
*Slc36a1*	Proton-coupled neutral amino acid transporter 1, l-glycine, l-proline	2.4	97	136
*Slc36a4*	Proton-coupled amino acid transporter 4	2.3	718	969
*Slc7a10*	Asc-type neutral amino acid transporter 1, d-serine, l-serine	2.1	783	935

*Expression levels of glucose and monocarboxylate transporters E15 and adult lateral ventricular choroid plexus. RNA-Seq analysis. Fold change (FC). Raw counts are transcript counts from original data. Raw count data have not been normalized and thus the calculated fold changes FC may be different from E/A raw count ratios. E is embryo only. Data on blood-brain amino acid transport in neonatal and adult brain. Rat: (Sershen and Lajtha, 1976; Baños et al., 1978; Lefauconnier and Trouvé, 1983; Benrabh and Lefauconnier, 1996); blood-CSF transport, (Al-Sarraf et al., 1995, 1997; Al-Sarraf, 2002). Rabbit: (Braun et al., 1980; Cornford et al., 1982; Cornford and Cornford, 1986). Data on blood-brain amino acid transport in adult brain. (Davson and Segal, 1996; Segal, 2001)*.

#### Divalent metal transporters

It has been known for many years that zinc is important for normal brain development and for function in the adult (Sandstead, 1985; Marger et al., 2014; Tyszka-Czochara et al., 2014). In the present study there were 4 zinc transporter genes that were expressed at a higher level in embryonic choroid plexus than in the adult in the fold range of 2.5–44 (Table 6). In the adult there were more up-regulated zinc transporter genes (6), but their expression level was generally much lower (2.7–12 fold). Both of the major zinc transporter families (*Slc30*, ZnT, and *Slc39*, ZIP) are represented in our dataset. The members of the two families function in opposite directions, thus maintaining cellular zinc homeostasis. ZnT proteins of Slc30 genes efflux zinc whereas ZIP proteins function to increase zinc uptake when cellular zinc is depleted (Marger et al., 2014; Tepaamorndech et al., 2014). Ten *Slc30* genes were identified in the present study (two higher in the embryo, *Slc30a2*, 44 FC; *Slc30a10*, 35 FC and four in the adult, *Slc30a3-6*, with only modest 2.7–4.5 FC). Ten *Slc39* genes were identified; *Slc39a8* was 37 FC higher in embryo. Three other members, Slc39a1, *Slc39a12*, and *Slc39a13* (FC 12, 12 and 3.9 respectively), were higher in adult (Table 6).

**Table 6:** **Expression of zinc and other metal transporters in embryonic (E15) and adult rat choroid plexus**.

**Gene name**	**Gene description**	**FC**	**Raw transcript counts**	
			**EMBRYO (E15)**	**ADULT**
**EMBRYO**				
**ZINC**		**E/A**		
*Slc30a2*	Zinc transporter 2, ZnT2	E	608	8
*Slc39a8*	Zinc transporter ZIP8	37	1493	24
*Slc30a10*	Zinc transporter 10, ZnT8, ZnT10	E	224	4
*Slc39a10*	Zinc transporter, 10 (Predicted), ZIP10	2.5	5547	1304
**OTHER METALS**				
*Sfxn1*	Sideroflexin-1, iron transport	14	1103	45
*Slc11a1*	Natural resistance-associated macrophage protein 1, Mn+, Cd+ transporter	E	130	8
*Heph*	Hephaestin, iron, copper transport	E	67	5
*Slc34a2*	Sodium-dependent phosphate transport protein 2B	6.1	107	10
*LOC100360087*	Ferritin light chain 1-like, iron transport	3.6	837	132
*Tfrc*	Transferrin receptor protein 1	3.1	1354	258
**ADULT**				
**ZINC**		**A/E**		
*Slc39a1*	ZIP1, zinc transport	12	2143	17839
*Slc39a12*	Zinc transporter ZIP12	12	489	3318
*Slc30a4*	Zinc transporter 4, ZnT4	4.5	2025	5248
*Slc39a13*	Zinc transporter ZIP13	3.9	758	1681
*Slc30a3*	Zinc transporter 3, ZnT3	3.8	54	117
*Slc30a5*	Zinc transporter 5, Co, ZnT5	3.4	1397	2719
*Slc30a6*	Zinc transporter 6, Co, ZnT6	2.7	491	752
**OTHER METALS**				
*Tf*	Transferrin, iron transport	183	2027	212407
*Lcn2*	Lipocalin 2, Signal recognition particle receptor subunit beta, iron transport	55	12	365
*Cp*	Ceruloplasmin copper transport	6.2	188	675
*Slc31a1*	High affinity copper uptake protein 1	4.6	6673	17839
*Slc20a1*	Sodium-dependent phosphate transporter 1	2.4	2155	2919
*Slc41a1*	MgtE-like magnesium transporter, member 1	2.3	401	530
**NO CHANGE WITH AGE**				
**ZINC**				
*Slc30a1*	ZNT1, zinc transport	-	1048	843
*Slc30a6*	ZNT6, zinc transport	-	491	752
*Slc30a7*	ZNT7, zinc transport	-	424	400
*Slc30a9*	ZNT9, zinc transport	-	1512	1526
*Slc39a3*	ZIP3 zinc transport	-	322	315
*Slc39a4*	ZIP4, zinc transport	-	58	38
*Slc39a5*	ZIP5, zinc transport	-	233	172
*Slc39a6*	LIV1, zinc transporter	-	1819	1241
*Slc39a7*	ZIP7, zinc transporter	-	4792	4670
*Slc39a9*	ZIP9, zinc transporter	-	798	540
*Slc11a2*	DMT1, divalent ion transport, including iron	-	1929	1518
**OTHER METALS**				
*Slc40a1*	iron-regulated transporter	-	889	455
*Slc31a2*	CTR2, copper transport	-	63	144
*Slc41a2*	magnesium transporter	-	20	63

*Expression levels of Zn and other metal transporters comparing E15 embryo and adult lateral ventricular choroid plexus obtained from RNA-Seq analysis. Fold change (FC), Embryo only (E). Raw counts are transcript counts from original data. Raw count data have not been normalized and thus the calculated fold changes FC may be different from E/A raw count ratios*.

The transferrin gene, *Tf*, was expressed in terms of transcript numbers at a high level in both the embryo and adult (Table 6), but much higher in the latter (183 fold), with a continuous pre-and post natal increase from embryo to adult stages. The transferrin receptor was expressed at a higher level in embryonic choroid plexus (3.1 fold) with intermediate expression levels at the perinatal stage of development. As indicated in Table 6 several other iron carriers were expressed at a higher level in the embryo than in the adult: sideroflexin-1, *Sfxn1* (14 fold), ferritin light chain 1-like (*LOC100360087*), 3.6 fold), and this difference in expression was maintained perinatally.

*Slc11a1* was expressed at much higher levels in the embryonic choroid plexus than in the adult (8.9 fold), and at still higher level at birth, while *Slc11a2* (DMT1) was found expressed at a similar level at all ages examined by both RNA-Seq and microarray approaches (Table 6). Several members of these SLC families of divalent metal transporters have been implicated in iron and manganese transport. Their expression in developing choroid plexus correlates with the higher uptake of manganese by postnatal choroid plexus cells *in vitro* as shown by Schmitt et al. (2011), and is discussed further below.

### Immunohistochemistry

Immunohistochemical investigations of the distribution of some efflux transporter proteins selected from the gene expression data included five enriched in the fetus: SLC 16a10 (TAT1), SLC 38a5 (SNAT5), SLC 4a1 (AE1), SLC 11a1 (NRAMP), SLC 1a3 (EAAT1), one enriched in the adult: SLC 5a5 (NIS) and one expressed at a low level in both fetal and adult choroid plexus: SLC 39a4 (ZIP4).

**Figure 4:**
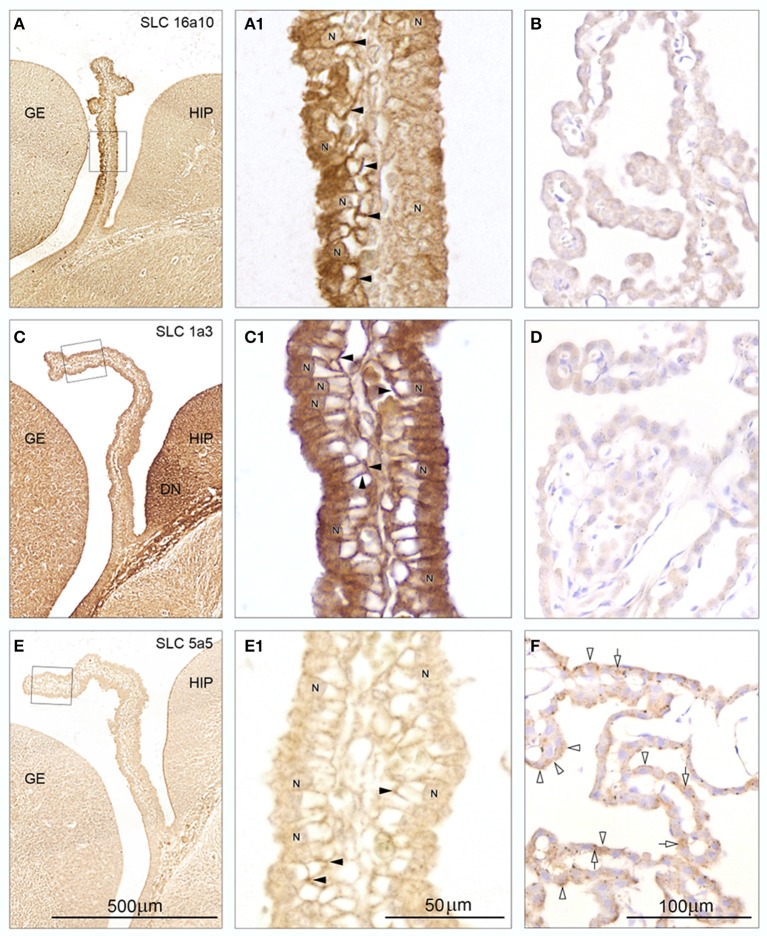
**Immunohistochemistry of influx transporters in sagittal and coronal sections from E15 and adult rat choroid plexus: SLC 16a10 (A,A1,B), SLC 1a3 (C,C1,D), and SLC 5a5 (E,E1,F)**. The *rectangles* in (**A,C,E)** from E15 are shown at higher magnification in **A1,C1,E1**. For comparison adult choroid plexus stained for the same three transporters is shown in **B,D,F**. Note the differences in distribution of immunoreactivity between E15 stained for the three different transporters and between E15 and adult choroid plexus stained for a given transporter. **(A)** The rostral-most leaflet of the lateral ventricular choroid plexus facing the ganglionic eminence (GE) demonstrates a very strong reactivity in contrast to the caudal-most leaflet facing the developing hippocampus (HIP). **(A1)** At higher magnification the Immunostaining fills the entire cytoplasm apical to the nuclei (N) and down along the basolateral membrane (*arrowheads*) just sparing the empty glycogen space in the epithelial cells of the rostral leaflet. The caudal leaflet is also positively stained and shows a more uniform cytoplasmic reactivity although condensed at the perinuclear membrane. **(B)** Adult choroid plexus shows virtually no immunostaining for SLC 16a10. **(C)** Both leaflets of the choroid plexus demonstrate a uniform cytoplasmic immunoreactivity for SLC 1a3 although not as strong as that of the adjacent dentate neuroepithelium (DN). **(C1)** At higher magnification the immunostaining of the entire plexus shows a pattern similar to that described for the rostral leaflet stained for SLC 16a10 (compare with **A1**). **(D)** Adult choroid plexus shows virtually no immunostaining for SLC 1a3. **(E)** The E15 choroid plexus shows very little immunoreactivity following staining for SLC 5a5 which on the other hand outlines the CSF surface of the hippocampus. **(E1)** At higher magnification a weak Immunostaining is localized to some basolateral cell membranes (*arrowheads*) of the choroid plexus epithelial cells. **(F)** Adult choroid plexus epithelial cells exhibit a uniform very fine granular cytoplasmic reactivity following immunostaining for SLC 5a5. Many cells also exhibit a coarse granular staining (*open arrows*) and apical membrane reactivity (*open arrowheads*). **(A,C,E)** Same magnification, *scale bar* 500 μm. (**A1,C1,E1**) Same magnification, scale bar 50 μm. (**B,D,F**) Same magnification, *scale bar* 100 μm.

The seven tested antibodies resulted in 3 different patterns of immunoreactivity in E15 plexus epithelial cells (“differential immunoreactivity, regional distribution-type,” “strong immunoreactivity, uniform distribution-type,” “weak immunoreactivity, uniform distribution-type”) and in 2 different patterns of immunoreactivity in adult plexus epithelial cells (“weak or lack of immunoreactivity,” “strong immunoreactivity”). Representative micrographs from the 5 different patterns of immunoreactivity are shown in Figure 4.

#### SLC 16a10

An example of “differential immunoreactivity, regional distribution-type” is shown in E15 and of “weak or lack of immunoreactivity” is presented in adult plexus. The embryonic plexus epithelial cell layer in the “plexus-fold” next to the ganglionic eminence but opposite to the developing hippocampus showed a stronger immunostaining than the “plexus-fold” facing the hippocampus (Figure 4A). At high magnification the prominent reactivity in the epithelial cell layer facing the ganglionic eminence was shown to be due to a combination of a marked fine granular reactivity in apical cytoplasm and along both apical and basolateral cell membranes, which were particularly strongly immunostained (*arrowheads* in Figure 4A1). In contrast the epithelial cell layer facing the hippocampus showed a more uniform immunoreactivity both in cytoplasm and along cell membranes. Adult choroid plexus epithelial cells exhibited virtually no immunostaining of either membranes or cytoplasm (Figure 4B).

#### SLC1a3

This protein showed an example of “strong immunoreactivity,” uniform distribution type at E15 and of “weak or lack of immunoreactivity” in adult plexus. Both embryonic plexus epithelial cell layers of the “plexus-fold”—the leaflet next to the ganglionic eminence and the other next to the developing hippocampus - showed a strong uniform cytoplasmic immunostaining (Figure 4C). At high magnification the prominent reactivity was shown to be due to a combination of a marked fine granular reactivity in apical cytoplasm and along both apical and basolateral cell membranes, which were particularly strongly immunostained (*arrowheads* in Figure 4C1) comparable to those stained for SLC16a10 in the plexus fold next to the ganglionic eminence shown in Figure 4A1. Adult choroid plexus epithelial cells showed virtually no cytoplasmic immunostaining and a lack of apical and basolateral cell membrane immunoreactivity (Figure 4D).

#### SLC5a5

Shown as an example of “weak immunoreactivity,” uniform distribution at E15 and of “strong immunoreactivity” in adult plexus. Embryonic plexus epithelial cells showed no cytoplasmic reactivity and no immunostaining of the apical cell membrane in marked contrast to the adjacent strongly stained CSF-hippocampal interface (Figure 4E). A weak basolateral cell membrane staining of plexus epithelial cells could be identified at high magnification (*arrowheads* in Figure 4E1). Adult choroid plexus exhibited a uniform fine and coarse granular cytoplasmic staining of most epithelial cells (*open arrows* in Figure 4F) and a distinct apical membrane staining (*arrowheads* in Figure 4F).

#### SLC39a4 (ZIP4), SLC38a5 (SNAT5), SLC4a1 (AE1), SLC11a1 (NRAMP)

Following immunostaining for SLC39a4 plexus epithelial cells were characterized by “weak immunoreactivity,” uniformly distributed in both E15 and adult plexus, whereas staining for SLC38a5, SLC4a1, and SLC11a1 resulted in immunoreactivity of the uniform distribution-type, however stronger along the basal and basolateral cell membrane in E15, and very little cytoplasmic staining and virtually no basolateral or apical membrane staining in adult plexus apart from SLC38a5 which showed a patchy immunostaining of the apical cell membrane (data not shown).

## Discussion

All but four of the 52 known families of SCL transporters were represented in the dataset from RNA sequencing of lateral ventricular choroid plexuses in the rat embryo (E15) and in the adult (Supplementary Table S1). Of the 389 individual SCLs, 64% had transcript numbers above the threshold taken to indicate likely functional significance (>100 sequence reads). 81 of these were expressed 2 fold higher or more in the embryonic plexus compared to the adult, 107 were expressed higher in the adult and 62 were expressed at similar levels at the two ages. Some genes were identifiable with transcript counts of 10–100, but thought less likely to be of functional significance; they have nonetheless been included in Tables 4–6. The results for microarray were generally consistent with those for RNA-Seq in their developmental profile.

It is apparent from the above analysis that many genes identified are from families involved in transport of ions important for homeostatic mechanisms in the CSF and interstitial fluid of the brain (Tables 2, 3, and Supplementary Tables S2, S3). The monovalent inorganic ion transporters known to be functionally active in adult choroid plexus and involved in CSF composition and secretion have been described in detail and their expression levels correlated with functional data in the paper by Liddelow et al. (2013). Divalent metal ions such as Ca^2+^, Zn^2+^, and Mg^2+^ may be involved in specific cellular functions in brain such a synaptic transmission of nerve impulses and are considered briefly below.

The immunohistochemical data presented above indicate that at least some of the most highly expressed *Slc* genes are able to produce their protein products. A more difficult problem is to establish the functional effectiveness of individual *Slc*s for example for glucose, particular amino acids or specific monocarboxylates. This is because of the overlap in substrates between many SLCs (see Tables 2, 3). Because of this and of upregulation of unaffected genes, even selective knockouts of single genes may not reveal the contribution of that particular gene to the transport of specific substrates (see Keep and Smith, 2011). However, it is possible to suggest that at least some of the genes are indeed functionally more active in embryonic choroid plexus by examining results of transport studies for glucose, amino acids, monocarboxylates, and divalent cations in developing brain.

### Glucose transport

The best known and ubiquitously distributed glucose transporter *Slc2a1* (GLUT1) was expressed at a 3-fold higher level in the embryonic choroid plexus at transcript levels (Table 4) suggesting that it would have significant functional activity at both ages, although higher in the embryo. It has been identified in developing choroid plexus in a number of previous studies (Vannucci, 1994; Baud et al., 2003); the level was highest during gestation followed by a 50% decline by P1 and a further decline during the first postnatal week (Bauer, 1998). Bauer (1998) suggests this reflects a decline in cerebral glycogen content (Kohle and Vannucci, 1977). Glycogen is particularly prominent in choroid plexus epithelial cells in the late embryonic and early neonatal period, after which it disappears (Schachenmayr, 1967 and see Netsky and Shuangshoti, 1975). *Slc2a3* (GLUT3) in the present study was expressed only at E15 in RNA-Seq compared to adult and in microarray was below adult at E19 and P2. The protein product of this gene was not identified in a previous study of postnatal rat choroid plexus (Vannucci, 1994). *Slc2a4* (GLUT4) was also expressed only in the embryo, although at a low transcript count (90). This appears to be consistent with the finding of Vannucci et al. (2000) in mouse choroid plexus where it was detected as early as at E14. In addition, in the present study *Slc5a10* (SGLT5), a sodium-glucose transporter was expressed exclusively in the embryonic choroid plexus. Previously it has only been identified in kidney cortex (Wright, 2013). However, the raw transcript count for this gene was low, thus its functional significance is unclear.

### Monocarboxylate transport

It has been long accepted that although glucose is a fundamentally important energy substrate in the brain, during development the brain also uses lactate and other monocarboxylates to a substantial degree. In the postnatal period in rodents it has been suggested that a switch occurs from a combination of glucose, lactate, and ketone body metabolism in the immature brain to a reliance on glucose in the adult (Cremer, 1982; Nehlig, 1997). Several monocarboxylate transporters have been found in the developing brain of rodents. *Slc16a1* (MCT1) is expressed and its protein product present in cerebrovascular endothelial cells of rodents at higher levels in early postnatal ages compared to later (Leino et al., 1999; Vannucci and Simpson, 2003). In contrast the level of *Slc16a7* (MCT2) expression and protein product, present predominantly in neuronal cells, do not appear to be developmentally regulated (Vannucci and Simpson, 2003). *Slc16a1* (MCT1), but not *Slc16a7* (MCT2), has been identified in adult mouse choroid plexus (Koehler-Stec et al. (1998).

The expression of monocarboxylate transporters appears to have been less studied in the embryonic brain barrier interfaces. Apart from a high expression of *Slc16a10* (MCT10) in embryonic mouse choroid plexus (Liddelow et al., 2012) nothing seems to be known about monocarboxylate transporters in the embryonic choroid plexus. This high expression in embryonic mouse choroid plexus is confirmed in the present study for embryonic rat choroid plexus. This is a particularly noteworthy finding because MCT10 is a transporter for tri-iodothyronine, T3 and thyroxine, T4 (Porterfield and Hendrick, 1992), which are essential for normal brain development. Inadequate delivery of T4 to the developing brain, usually due to iodine deficiency, results in cretinism (Rivas and Naranjo, 2007; Skeaff, 2011). Given the prominence of the choroid plexuses in the embryonic brain compared to vascularisation (Johansson et al., 2008), it may be that MCT10 in the choroid plexuses along with TTR, a thyroid hormone carrier highly expressed throughout development, is the major mechanism by which thyroxine is delivered to the brain in early stages of its development. This is also in line with the low developmental expression of *Slco1c1*, the main thyroid hormone transporter expressed at the blood-brain interfaces in the adult (Kratzer et al., 2013).

Published data demonstrating at the protein level, that some of the glucose transporters (Vannucci, 1994; Bauer, 1998; Baud et al., 2003) and monocarboxylate transporters (Leino et al., 1999; Vannucci and Simpson, 2003) are present in the choroid plexus barrier interface between blood and CSF in the developing brain indicate that these transporters are probably functional. In addition, it has been shown that brain uptake of both glucose (Braun et al., 1980; Cornford and Cornford, 1986) and lactate (Cornford et al., 1982; Cornford and Cornford, 1986) is greater in newborn rabbits than in adults, which again supports the likelihood that transporters for these metabolically important nutrients are indeed functionally more active in the developing brain. It is not clear from the transport studies, which specific glucose transporters would have been involved, but the transcript numbers perhaps give some indication of the relative functional importance of the different transporters. The most highly expressed glucose transporter at both ages was *Slc2a12*, although 5 fold more in the adult.

The finding in the present study of a high expression of glucose transporters in the embryonic brain, but not of those transporting lactate and other monocarboxylates, suggests that the embryonic brain is like the adult brain in being exclusively dependent on glucose metabolism as a source of energy, as suggested previously by Vannucci and Vannucci (2000) and unlike the postnatal brain in which monocarboxylate transport should be functionally significant. However, of all the monocarboxylate transporters detected on the arrays, three genes, *Slc16a3*, *Slc16a4* (not detected by RNA-Seq) and *Slc16a10*, were expressed at a higher level at E19 than at P2 and adult, and the others were expressed at a higher level in adult. It is possible that a transient up-regulation occurs later in post-natal development for some of these transporters. This was shown for *Slc16a1* (MCT1) in the postnatal period at least in rodents, and may relate to the high fat content in human and rodent maternal milk, which generates substantial ketone body concentrations in blood, as a consequence of liver metabolism (Vannucci and Vannucci, 2000; Vannucci and Simpson, 2003).

### Amino acid transport

There is a limited amount of information concerning the effectiveness of amino acid transport *in vivo*, that could support expression data. Experiments mainly carried out in the 1970s-80s were mostly performed in neonatal rats (e.g., Baños et al., 1978; Lefauconnier and Trouvé, 1983, see Table 6) but some were in fetal and postnatal rabbits (Braun et al., 1980; Cornford et al., 1982; Cornford and Cornford, 1986). Very few of these studies estimated amino acid entry from blood into CSF. Most studied brain tissue; an exception is the papers of Al-Sarraf and colleagues (Al-Sarraf et al., 1995, 1997; Al-Sarraf, 2002). However, given the paucity of blood vessels in very immature brain it is likely that much of the amino acid detected in brain tissue of, for example neonatal rodent, arrived there via the choroid plexuses and CSF. Another interpretational difficulty is that these experiments were done before data on individual transporters were available. Thus, at best we can only compare individual or classes of amino acids shown to be transported *in vivo* in the developing brain with the various families of SLC transporters known to transport individual or classes of amino acids (Table 5). However, the finding (described in the papers cited above) that transport of many amino acids in developing brain is greater than in the adult strongly supports the proposition that the higher expression of many amino acid transporters in the developing brain is indeed reflected in greater transport activity.

### Metal transport

Both of the major zinc transporter families (*Slc30*, ZnT and *Slc39*, ZIP) are represented in our two datasets. The members of the two families function in opposite directions, thus maintaining cellular zinc homeostasis. ZnT proteins of *Slc30* gene efflux zinc whereas ZIP proteins function to increase zinc uptake when cellular zinc is depleted (Marger et al., 2014; Tepaamorndech et al., 2014). In the context of transfer of zinc across the epithelial cells of the choroid plexus it may be that they operate in tandem. Support for this comes from the observation that SCL39A6 (LIV-1) has been immunolocalised to the apical membrane of the neonatal choroid plexus epithelial cells (Chowanadisai et al., 2005).

None of the six *Slc30* genes identified in this study (two high in the embryo, *Slc30a2*, *Slc30a10*, and four in the adult, *Slc30a3-6*, 2.7–4.5 FC) have previously been reported in choroid plexus. Of the three *Slc39a* genes identified (*Slc39a8*, 37 FC higher in embryo; *Slc39a12* and *Slc39a13*, FC 12 and 3.9 higher in adult, respectively) none appear to have been identified previously in choroid plexus. The microarray data show that *Slc30* and *Slc39* genes identified in E15, but not in adult, are still expressed at the perinatal stages. Other genes of these families whose expression is lower at E15 display a rapid developmental increase in expression, to reach adult level shortly after birth. Altogether this suggests that choroidal zinc transport is substantial throughout development.

Zinc is an essential nutrient for many cellular biochemical processes (Tyszka-Czochara et al., 2014) and is essential for normal brain development and function (Sandstead, 1985). The requirement for zinc is increased during embryonic development and deficiency results in birth defects (McKenzie et al., 1975; Sandstead et al., 1975), including multiple defects in neuronal development, by mechanisms that are unclear (Chowanadisai et al., 2013a; Grabrucker et al., 2014). *Slc39a12* (ZIP12) has been found to be highly expressed in brain of human and mouse adults and in development appears to be involved in early stages of neuronal differentiation and neurite outgrowth, through its role as a zinc transporter (Chowanadisai et al., 2013a,b). Zinc has specific functions in synaptic transmission. 5–15% of the brain's zinc content is concentrated in synaptic vesicles of glutamatergic neurons. This is of importance for neuronal function in the developing and adult brain, but the identification of several genes for glutamate receptors and other transmitters, as reported earlier (Liddelow et al., 2013) suggests that the zinc transporters in choroid plexus may have a function in relation to the innervation of the choroid plexus. A more general consideration of the importance for neural function in the brain is outside the scope of this paper. Finally, zinc transporters, like other metal transporters, may transport this metal for use by the epithelial cells themselves, as discussed below.

Iron uptake into the brain occurs across both the blood-brain and blood-CSF barriers (Moos and Morgan, 2000). Transferrin, which binds iron, makes an important contribution at both interfaces, but there are some differences in the mechanisms involved. At the blood-brain barrier, transferrin receptors on the cerebral endothelial cells bind transferrin-carrying iron, which is taken up into the cells. This mechanism and subsequent steps in iron delivery to the brain have been described in detail (e.g., Leitner and Connor, 2012; Singh et al., 2014) and will not be considered further here. However, the uptake of both iron and transferrin is greater in the developing brain, the more so for iron itself (Morgan and Moos, 2002). The involvement of the choroid plexuses in iron transport into the brain in addition to transferrin uptake via transferrin receptors also involves the synthesis and secretion of transferrin into the CSF (Leitner and Connor, 2012). In the adult brain it has been suggested that the choroid plexuses, compared to the cerebral vasculature may play a much larger role in iron uptake than previously thought (Rouault et al., 2009). As mentioned above in relation to other transport properties, the early development of the choroid plexuses compared to brain vasculature (Johansson et al., 2008) indicates that the plexuses are likely to be even more important for iron transport in the developing brain. The likely importance of the transferrin gene, *Tf*, for iron transport in both the embryonic and adult choroid plexus is emphasized by the high transcript numbers (Table 6), although much higher in the adult (183 fold). *Tf* expression however increases rapidly, and is only 5- and 2.3 times lower at E19 and P2 respectively than in adult. Several other iron transporters were expressed at a higher level in the embryo than in the adult: sideroflexin-1, *Sfxn1* and ferritin light chain 1-like (*LOC100360087*), but in terms of transcripts both were expressed at lower levels than *Tf* (Table 6). *Slc11a1* and *hephaestin* were only expressed in the embryonic plexus but at low levels, so their functional contribution is doubtful. The transferrin receptor was expressed at a higher level in embryonic choroid plexus (Table 6) but this was reversed at birth. Several studies have described developmentally regulated immunohistochemical staining for several of the iron transporters in the choroid plexus (e.g., Siddappa et al., 2003; Wang et al., 2008). Moos and Morgan (1998) have provided direct evidence for the transfer of ^59^Fe from blood into brain and CSF. The importance for iron in brain function lies in its involvement in cellular respiration and cell proliferation during development. The brain also requires iron for myelination and neurotransmission (Leitner and Connor, 2012).

Manganese (Mn) is an essential biological element and a necessary cofactor in a number of important enzymatic reactions (Gunter et al., 2013) and is an essential micronutrient for normal brain development (Santamaria and Sulsky, 2010). Manganese transport has been demonstrated in choroid plexus freshly isolated from P2 and adult rats; uptake into plexus cells was found to be greater in P2 choroid plexuses. Unidirectional blood-to-CSF transport of manganese was demonstrated using P2-derived *in vitro* reconstituted epithelium (Schmitt et al., 2011). As these authors point out it is not possible to link this transport to specific transporters but suggest the divalent metal transporters SLC11a2, SLC11a3, SLC39a8, and SCL39a14 as potential candidates. In the RNA-Seq and array screens in the present study *Slc11a1* (8.9 fold) and *Slc39a8* (37 fold) were expressed at higher levels in the embryonic and perinatal choroid plexus, which correlated with the higher uptake shown by Schmitt et al. (2011). In addition, transport of manganese by transferrin has been demonstrated (Gunter et al., 2013). Copper is an essential nutrient for normal growth and development of the fetus and neonate (Uriu-Adams et al., 2010). It is a co-factor for several enzymes and also a structural component with involvement in many physiological pathways in the brain; in particular it is a component of cytochrome c oxidase, a member of the super-family of heme-copper-containing oxidases (Scheiber et al., 2014). In addition to divalent ion transporters thought to transport copper that were identified in the present study (e.g., *Slc11a2*) at least two more specific copper transporters were expressed in the choroid plexus. Ceruloplasmin was expressed at modest transcript numbers some 6.2 fold higher in the adult plexus (Table 6). The high affinity copper transporter, *Slc31a1*, was amongst the highest expressed transporter genes at both ages identified in this study and was 4.6 fold higher in the adult (Table 6). These data are in accordance with *in situ* hybridization showing the expression of *Slc31a1* in choroid plexus and its rapid, choroid plexus-restricted increase from E14 to E18.5 in mice (Kuo et al., 2001). These authors suggest that the choroid plexus is a major pathway of copper supply to the developing brain.

Thus, genes for all of the main metal transporters have been identified in the choroid plexus, several of them expressed at higher levels in the embryo than in the adult, presumably reflecting the importance of their involvement in brain development. Metals are also needed for normal choroidal epithelial functions. Iron-containing cytochrome levels are high in choroid plexus as a result of high mitochondrial activity. Copper, zinc, and manganese are cofactors of several enzymatic systems, in particular superoxide dismutases (SOD) involved in regulating oxidative stress. Both Mn-SOD and Cu-Zn-SOD are well expressed in choroid plexus throughout development (Yon et al., 2008, 2011). Thus, a primary function of metal transporters in the choroid plexus may be to supply metals required for proper functioning of the blood-CSF barrier. Of note, copper deficiency leads to a specific increase in *Slc31a1* expression at the choroid plexus, a finding interpreted as a compensatory mechanism to spare the choroid plexus itself from copper deficiency (Gybina and Prohaska, 2006).

### Immunohistochemistry

The protein products and their cellular distribution were demonstrated in E15 and adult choroid plexus for a selection of the *Slc*s identified in the gene screens, using immunohistochemistry. Three patterns of immunostaining were identified in E15 plexus epithelial cells: (i) differential immunoreactivity with regional distribution (e.g., SLC16a10), (ii) strong immunoreactivity with uniform distribution (SLC1a3), and (iii) weak immunoreactivity with uniform distribution (SLC5a5). In the adult plexus two different patterns of immunoreactivity in epithelial cells were noted: (i) strong immunoreactivity (SLC5a5) and (ii) weak or lack of immunoreactivity (SLC1a3, SLC16a10, SLC39a4). In a previous study (Liddelow et al., 2013) we illustrated immunostaining for SLC4a1 that was strong and uniformly distributed in E15 rat choroid plexus. Immunostaining for AQP1 has also been shown to have a uniform cellular distribution which was strong in E15 rat choroid plexus with characteristic localization in the apical membrane of all epithelial cells with weaker staining in the basolateral membrane; in the adult plexus the immunostaining was even stronger (Johansson et al., 2005). There was good correspondence between gene expression level and strength of protein immunostaining (Table 7).

**Table 7:** **Comparison of expression level (transcript numbers) and immunostaining for protein gene product**.

**Gene ID**	**Protein ID**	**E15 transcript raw counts**	**E15 immunostaining**	**Adult transcript raw counts**	**Adult immunostaining**
*Slc16a10*	MCT10	3089	Strong, regional	51	Weak, uniform
*Slc38a5*	SNAT5	1021	Moderate, uniform	6	Minimal, few patches
*Slc4a1*	AE1^*^	7109	Strong, uniform	70	Minimal-absent
*Slc11a1*	NRAMP	130	Moderate, uniform	8	Absent
*Slc1a3*	EAAT1	4372	Strong, uniform	577	Weak, uniform
*Slc5a5*	NIS	163	Weak, uniform	4916	Strong, uniform
*Slc39a4*	ZIP4	58	Weak, uniform	38	Apical, membrane
*Aqp1*	AQP1^**^	20112	Strong, uniform	32149	Very strong, uniform

*Uniform indicates all cells were positive. Regional indicates staining confined to only some (contiguous) cells*.

* from Liddelow et al. (2013),

** *from Johansson et al. 2005)*.

## Limitations of study

There are several limitations in this study. One concerns the use of whole choroid plexus tissue. However, as discussed previously (Liddelow et al., 2013) the epithelium is the predominant cell type, representing up to 90% of the plexus tissue (Keep and Jones, 1990; Liddelow et al., 2012). The absence of transcript for Cldn5 (a brain barrier cerebral endothelial-specific claudin family member) in the current RNA-Seq dataset suggests that any possible contamination is below detectable limits (Liddelow et al., 2013). The immunohistochemical evidence in that paper and in the current study for a range of protein products of the genes identified in the choroid plexus samples showed that these proteins were largely confined to the epithelial cells of the plexus. The analysis of large RNA sequencing databases is still quite new and relies on the accuracy of the annotations for each species studied. The rat genome is well annotated and the number of unknown genes is very low, however, there is still the possibility that some transcripts were missed. The papers based on microarray studies of adult mouse choroid plexus (Marques et al., 2011 and Janssen et al., 2013) on adult human and mouse adult choroid plexus provide important additional data on gene expression in the choroid plexuses of other species. These together with that of Liddelow et al. (2012) a mouse microarray study and that of Ek et al. (2015) an RNA-Seq study in primate choroid plexus provide comprehensive datasets for interspecies comparison reviews; however, this is outside the scope of this research paper.

We did not attempt to analyze all functional groups represented in the transcriptomic dataset. For example *Slc16a10* showed strikingly high expression (Table 4) exclusively in the embryo; it has been included primarily because it is a monocarboxylate transporter (MCT10) although its little investigated putative function as a thyroid hormone transporter may well prove to be more functionally important. This high expression in the embryonic choroid plexus is in striking contrast to the expression of the best-known thyroxine transporter, *Slc16a2*, which barely changed expression during development.

Another limitation is that, as pointed out above, expression data alone do not necessarily equate to functional activity. The immunohistochemical evidence in this paper, in Liddelow et al. (2013) and in earlier reports summarized in the Discussion shows that at least some of the mRNAs are being translated to protein. The summary of *in vivo* physiological transport studies for glucose, amino acids, monocarboxylates and metals in developing brain show a good correlation with the level of expression of relevant transporter genes, although because of overlap and redundancy it is generally not possible to pinpoint the specific contribution of any one gene.

## Conclusions

The results from this study and two earlier ones on gene expression in embryonic and adult rat choroid plexus (Kratzer et al., 2013; Liddelow et al., 2013) as well as a study of embryonic and adult mouse choroid plexus (Liddelow et al., 2012) show that a very large number of transporter genes are expressed in the embryonic lateral ventricular choroid plexuses as early as E15 which is only a day after these plexuses appear in the rat brain (Johansson et al., 2005). In the present study 64% of all the 389 known SLC transporters, with representatives of all but 4 of the 52 SLC families, were identified. Many of these were expressed at higher or similar levels in the embryonic compared to the adult plexus. This suggests that the lateral ventricular choroid plexuses make an important contribution to supply of nutrients and micronutrients to the brain early in development. This conclusion is supported by the results of immunohistochemistry for a number of the protein products of genes expressed in the embryonic and adult choroid plexus (Figure 4), as well as data from published transport studies for glucose, monocarboxylates, amino acids and divalent metals summarized in the Discussion.

## Author contributions

All of the listed authors contributed to the conception, design, research, drafting and final approval of the work. They each agree to be accountable for all aspects of the work.

### Conflict of interest statement

The authors declare that the research was conducted in the absence of any commercial or financial relationships that could be construed as a potential conflict of interest.

## Abbreviations

E: embryonic day
CSF: cerebrospinal fluid.
